# The Oncogenic Functions of Nicotinic Acetylcholine Receptors

**DOI:** 10.1155/2016/9650481

**Published:** 2016-02-14

**Authors:** Yue Zhao

**Affiliations:** Center of Cell biology and Cancer Research, Albany Medical College, 47 New Scotland Avenue, Albany, NY 12208, USA

## Abstract

Nicotinic acetylcholine receptors (nAChRs) are ion channels that are expressed in the cell membrane of all mammalian cells, including cancer cells. Recent findings suggest that nAChRs not only mediate nicotine addiction in the brain but also contribute to the development and progression of cancers directly induced by nicotine and its derived carcinogenic nitrosamines whereas deregulation of the nAChRs is observed in many cancers, and genome-wide association studies (GWAS) indicate that SNPs nAChRs associate with risks of lung cancers and nicotine addiction. Emerging evidences suggest nAChRs are posited at the central regulatory loops of numerous cell growth and prosurvival signal pathways and also mediate the synthesis and release of stimulatory and inhibitory neurotransmitters induced by their agonists. Thus nAChRs mediated cell signaling plays an important role in stimulating the growth and angiogenic and neurogenic factors and mediating oncogenic signal transduction during cancer development in a cell type specific manner. In this review, we provide an integrated view of nAChRs signaling in cancer, heightening on the oncogenic properties of nAChRs that may be targeted for cancer treatment.

## 1. Introduction

The nicotinic acetylcholine receptors (nAChRs) are of a family of ligands gated ion channels that are expressed in the cell membrane of all mammalian cells, including cancer cells [1]. In the nervous system nAChRs have high permeability to calcium, modulated by the extracellular calcium concentrations, phosphorylated by calcium-dependent serine/threonine kinases to regulate the release and activation of neuronal transmitters [2–5]. nAChRs are known to play several important roles involved in learning and cognition through regulating of synaptic plasticity, neuronal growth, differentiation, and survival [6]. The discovery of their expression on nonneuronal cells implicates their broad biological functions involved in cell proliferation, apoptosis, migration, and signal transduction. Recent findings suggest the imbalanced expressions of different subtypes of nAChRs in the cells contribute to the pathogenesis of diseases such as cancer [7].

Cigarette smoking or environmental tobacco smoke is an important risk factor for many types of cancers, including lung cancer, oral cancer, laryngeal cancer, oropharyngeal/hypopharyngeal caner, esophageal cancer, gastric cancer, liver cancer, pancreatic cancer, bladder cancer, renal cancer, cervical carcinoma, myeloid leukaemia, and colorectal cancer [8]. Among the carcinogens presented in tobacco, nicotine acts on nAChRs in the central nervous system (CNS) and causes addiction to smoke [9]. And two of its metabolites, namely, 4-(methylnitrosamino)-1-(3-pyridyl)-1-butanone (NNK) and N-nitrosonornicotine (NNN), bind to nicotinic receptor with much higher affinity than that of nicotine [7]. Recent studies indicated nicotine is able to induce cancer directly via promoting proliferation, inhibiting apoptosis of cancer cells, and stimulating tumor angiogenesis. These findings suggest that nAChRs are the central regulatory module of multiple downstream oncogenic signaling pathways in mediating the cellular responses of nicotine and its derivatives [8]. And nAChRs mediated effects of nicotine function in coalition with the mutagenic effects of the cancerogenic nitrosamine derivatives and reactive oxygen species activated by intracellular nicotine to promote tumor development and progression in tobacco related cancers.

**Figure 1:**
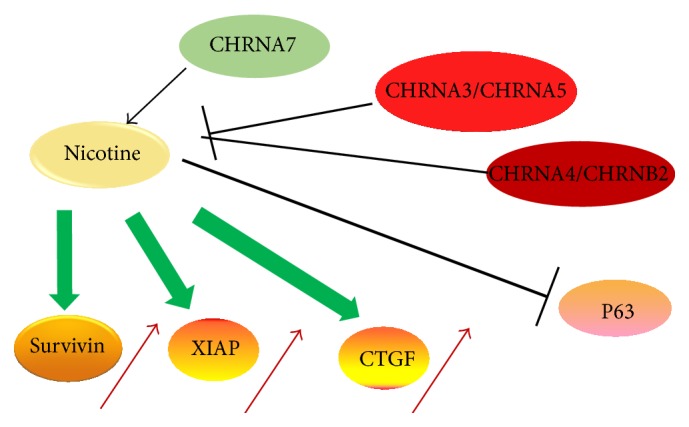
Differential effects of different nAChR subtypes on cell growth [21, 70, 96].

The nAChRs can either be composed of five identical *α*7, *α*8, or *α*9 subunits (homomeric nAChRs) or consist of combinations of *α*2–*α*6 or *α*10 subunits with *β*2–*β*4 subunits (heteromeric nAChRs). *α*7-nAChR and *α*4*β*2-nAChR are the evolutionarily oldest nAChRs predominantly expressed in the mammalian brain [10]. *α*7-nAChR is selective for Ca2+ and other nAChRs allowing the influx of different cations (Na+, K+, and Ca2+) [11, 12]. *α*7-nAChR is the most growth stimulatory nAChR in cancer cells, whereas *α*4*β*2-nAChR is the growth inhibitory receptor. Under normal physiological conditions, nicotine binds to *α*4*β*2-nAChR with higher affinity than *α*7-nAChRs. However, in smokers chronic exposure to nicotine or nicotine-derived carcinogenic nitrosamines leads to the upregulation of all nAChRs and long-term inactivation (or desensitization) of the 2*α*4*β*-nAChR [11, 13]; in contrast, the sensitivity of *α*7-nAChR remains unchanged [13]. Thus chronic exposure to nicotine causes selective activation of the cancer stimulatory nAChRs in the cell (Figure 1).

The affinity of NNK for *α*7-nAChR is 1,300 times higher than that of nicotine, whereas the affinity of NNN for heteromeric *α*–*β* nAChRs is 5,000 times higher than that of nicotine [14, 15]. Thus NNK and NNN can cause displacement of nicotine from these receptors as a result of their higher affinity for nAChRs. Therefore nitrosamines may cause many of the cardiovascular, neuropsychological, and cancer-stimulating effects similar to nicotine. Thus, nicotine, NNK, and NNN bind to nAChRs and other receptors, leading to activation of the serine/threonine kinase AKT, protein kinase A (PKA), and other factors [16, 17].

Based upon recent discoveries in the field, an increasing body of evidence suggests the positive correlations between nAChRs signaling and cancer incidences related to cigarette smoking. Particularly, lung cancers, pancreatic cancers, and esophageal cancers are among the most commonly induced cancers triggered by cigarette smoking and nAChR signaling [8]. In this review we have special focus on the genetic predisposition and molecular pathogenesis of cancers originated from these three organs in related nAChRs.

## 2. Genetic Variants of nAChRs in Association with Cancer

Single nucleotide polymorphisms (SNPs) of the chromosome* 15q25* region, which contains *α5-α3-β4* nAChR gene cluster (*CHRNA5-CHRNA3-CHRNB4*), is frequently associated with nicotine- (tobacco-) dependence, chronic obstructive pulmonary disease (COPD), and lung cancer in genome-wide association studies (GWAS) [18]. The association of the SNPs of* 15q25* genomic region with COPD and lung cancer could mediate by the combined effects of the oncogenic nAChR signaling and the neurological effects of nicotine addiction. Among these SNPs rs16969968 in* CHRNA5*, rs1051730 in* CHRNA3,* and rs8034191 are the most studied three SNPs of the region [18, 19].* CHRNA3* and* CHRNA5* are arranged in a tail-to-tail configuration on the opposite strand of the DNA, and the two variants rs1051730 and rs16969968 are in a complete linkage disequilibrium [*r* ^2^ = 0.98 in samples of Europeans/Caucasians]. Similarly rs1051370 is in strong linkage disequilibrium with rs8034191; thus some studies report the results for rs1051370 only. Notably, Chen et al. reported rs1051730 is associated with larger tumor size at diagnosis of squamous cell carcinoma. rs16969968 is a G-to-A [aspartic acid- (D-) to-asparagine (N)] missense variant at amino acid position 398 of* CHRNA5* [*α*5 (Asn398) D398N] [20]. And 398N is less potent than the variant 398D in protecting cells against the nicotine *α*7-nAChR mediated signaling making cells more susceptible to proliferation and migration [21]. Consistently, risk allele D-Asparagine is observed to reduce the function of *α*4*β*2*α*5-nAChR [18].

Alternatively, polymorphisms in linkage disequilibrium with rs16969968 may modulate the expression of* CHRNA5* [22, 23]. Thus the expression of functional (*α*3*β*2)2*α*5-nAChRS may play an important role in regulating the homeostasis and integrity of bronchial mucosa under physical, chemical, and immunological damage. Depending on the balanced regulation of the nAChRs, bronchial mucosa may undergo repair and recovery or give rise to precancerous lesion or hyperplasia when these receptors are deregulated. Moreover, NKK induced bronchial cell proliferation and the susceptibility to the tumorigenic transformation were reported to associate with different variants of human *α*9-nAChR subunit protein (S442 as the most frequent) [24]. Thus polymorphisms in* CHRNA5-CHRNA3-CHRNB4* gene cluster may modulate the dynamics of the normal bronchial epithelium under stress conditions to influence cancer risks [25]. Similarly, these SNPs associated with varied activity of nAChRs may associate with enhanced invasiveness and metastatic capacity. Besides, the effects of the* 15q25* polymorphism may impact on the neural behavioral effects on addiction to nicotine, resulting in an increased tobacco consumption, and so forth [26].

Interestingly Wu et al. reported rs8034191, rs1051730, and rs16969968 identified in previous GWAS are extremely rare in Asians, whereas they have identified four novel SNPs that were associated with significantly increased lung cancer risk and smoking behavior in Chinese population [27]. Particularly they have identified that rs6495309T>C considerably influenced the* CHRNA3* promoter activity, leading to higher *α*3-nAChR protein level and an increased risk of lung cancer. This seemingly contradictory observation could be explained as upregulation of (*α*3*β*2)2*α*5-nAChR in brain may dampen the nicotine responses mediated by *α*7-nAChR and consequently leads to reduced dopamine release upon nicotine induction [26]. Thus individuals with rs6495309C allele may need to consume more nicotine to reach the addictive neurological effects, leading to higher levels of exposure to smoking.

## 3. The Oncogenic Effects of Neurotransmitters Mediated by nAChRs

Stress neurotransmitters such as dopamine can stimulate the growth of cancer cells* in vitro*, which is in accord with nAChRs' role in regulating the release and synthesis of these neurotransmitters* in vivo* [13]. The effects are partly due to the facts that growth of nerve endings into the tumor microenvironment (neurogenesis) [28, 29] is necessary for the development of many cancers. The process is triggered by neurotrophic factors released from tumor cells to promote the nerve fibres growth into tumor tissues [30]. Consistently, *α*7-nAChR can promote neurogenesis by stimulating glutamate production whereas *α*4*β*2-nAChR can regulate neurogenesis by regulating Gamma-Amino Butyric Acid (GABA) synthesis and release [31, 32]. More importantly, the autocrine neurotransmitters of the catecholamine family play important roles in the carcinogenic pathways regulated by nAChRs. Thus under physiological conditions other risk factors also activate nAChRs to promote cancers in the body, such as psychological stress, and also activate the neuronal pathway through the activation of nAChRs and beta-adrenergic receptors [33].

Similarly NNK can stimulate the growth and migration of small airway epithelial cells through activation of *β*-adrenergic receptor which further transactivates EGFR through cAMP signaling [34–36]. *β*-adrenergic agonists such as adrenaline and noradrenaline triggered by nAChRs signaling are responsible for the development pulmonary adenocarcinomas (PACs). And adrenaline treated hamsters showed with significantly increased tumor growth in the NNK induced small-airway-derived PAC model [29]. Similarly noradrenaline plays an important role in promoting the growth of gastrointestinal cancer; it can mediate nicotine signaling through activation of ERK1-ERK2, cyclooxygenase 2 (COX2), prostaglandin E2 (PGE2), and VEGF [16, 37, 38]. Consistently, increased synthesis and releasing of noradrenaline and adrenaline are observed in colon cancer cells by nicotine treatment* in vitro*, an effect that is blocked by *α*7-nAChR antagonist [39]. Thus, the *β*-adrenergic signaling, transactivation of the EGFR, and releasing of EGF are the major contributors to the effects of tumor growth and angiogenesis mediated by nAChRs in colon cancer. Such an effect of nAChR signaling is also observed in many other types of cancers; for instance, the proliferation of mesothelioma cells is stimulated by nicotine through activation of the ERK1-ERK2 signaling cascade and nicotine also inhibits the apoptosis of the cell through activation of NF-*κ*B and phosphorylation of BAD [40]. In bladder cancer cells ERK1-ERK2 as well as STAT3 is also activated by nicotine through nAChRs and *β*-adrenergic receptors [41].

Suppressive neurotransmitters such as GABA also played a role in regulating cancer cell, and they are synthesized and released by cancer cells in an autocrine fashion. Researches indicated NNK can cause the decreased GABA level in PAC cells and further leads to decreased GABA dependent migration of PAC cells* in vitro* [42]. Desensitization of *α*4*β*2-nAChR is the major cause for decreased release of GABA in smokers and NNK treated hamsters [11, 13, 42]. Consistently, the RNA level of *α*4-nAChR has been observed to be significantly lower in PAC tissues than that of normal lung tissues [43]. Recent studies indicate suppressive neurotransmitter GABA can inhibit adrenaline induced migration of many types of cancer including colon cancer, prostate cancer, and breast cancer [44]. Joseph et al. reported the tumor suppressor function of GABA in lung adenocarcinoma [43]; similarly GABA can inhibit G*α*i-mediated inhibition of adenylyl cyclase and further leads to the inhibition of isoproterenol induced DNA synthesis and migration [45]. These findings are in accord with the association between increased releases of stress neurotransmitters caused by smoking and increased risk of PAC, which is caused by upregulation of *α*7-nAChR and a concomitant desensitization of *α*4*β*2-nAChR induced by smoking.

## 4. nAChRs in Regulating Tumor Angiogenesis

The pathological angiogenesis of tumor growth and metastasis induced by nicotine has been firstly reported by Heeschen et al. [46]. The proliferation of Lewis lung cancer cells which do not have functional nAChRs was not stimulated by nicotine* in vitro*. In contrast, accelerated tumor growth was observed after systemic administration of nicotine in xenograft mouse model [46]. And a 5-fold increase of capillary density in the tumor nodules was observed after nicotine administration. These findings suggest nicotine promotes tumor angiogenesis rather than affecting tumor cell proliferation directly in the Lewis lung cancer model. Later work showed second-hand smoke increased tumor angiogenesis and tumor growth, an effect that is associated with elevated plasma VEGF in the Lewis lung cancer model [47]. Consistently, increased endothelial progenitor cells were recruited to the ischemic sites in mice after nicotine administration [48].* In vitro* treatment of 10 nM nicotine to human endothelial progenitor cells increased the viability, migratory, and adhesive and vasculogenesis ability of these cells [49]. nAChRs antagonists mecamylamine and *α*-bungarotoxin can abolish the effect of nicotine on human endothelial progenitors [50].

Cholinergic angiogenesis is mainly mediated by *α*7-nAChR, which is predominantly expressed in the endothelial cell [50]. Other nAChRs modulate cholinergic angiogenesis through interacting with *α*7-nAChR. Notably, hypoxia can induce upregulation of *α*7-nAChR in endothelial cells. And ischemic hindlimb of the mouse expressed increased *α*7-nAChRs [50]. Consistently *α*7-nAChR antagonist *α*-bungarotoxin can suppress the increased endothelial cell migration, proliferation, and tube formation induced by nicotine* in vitro*. And the angiogenesis effects of nicotine are blunted in mice deficient with *α*7-nAChR [50]. Moreover, the effect of *α*7-nAChR on angiogenesis is further demonstrated by the *α*7-nAChR antagonist MG624 decrease of the angiogenesis effect of nicotine* in vitro* and in xenograft mouse model of small cell lung cancer. The effect of MG624 is probably mediated by inhibition of nicotine induced release of fibroblast growth factor 2 (FGF2) through activation of early growth response gene 1 [51]. Another research indicated that knockdown of *α*7-nAChR suppressed nicotine induced tubulogenesis of human retinal endothelial cells.

Other subunits of nAChRs are also expressed in the endothelia cells [52]. Interestingly, knockdown of* CHRNA9* in endothelial cells enhanced nicotine induced cell proliferation, migration, and tube formation [53]. The effect is probably caused by the compensatory increase of *α*7-nAChR on the cell membrane of endothelial cells.

The angiogenesis effect of nAChRs can function independently of exogenously added nicotine. Matrigel tube formation assay showed that nAChR antagonists have suppressive effects on angiogenesis [50]. Interestingly, antagonists of endothelial nAChR can also suppress the angiogenic processes of VEGF and FGF. These findings suggest pathways involved in nAChRs mediated signaling interact with the angiogenesis pathways of VEGF and FGF. And microarray studies indicated concordant transcriptional profiles induced by nicotine, VEGF, and FGF, which suggest angiogenic growth factors and cholinergic signaling pathways have close interactions [54]. In addition, endothelial cells can synthesize acetylcholine as an autocrine angiogenic factor [55, 56]. Besides acetylcholine, SLURP1/SLURP2 can also function as endogenous agonists of nAChR, and these proteins allosterically modify and activate nAChRs [57].

## 5. nAChRs Signaling in Lung Cancers

In pulmonary neuroendocrine cells (PNECs), nicotine or NNK stimulates the proliferation of PNECs* in vitro* through activation of protein kinase C (PKC), the serine/threonine kinase RAF1, the mitogen activated kinases ERK1 and ERK2, and the transcription factors FOS, JUN, and MYC. These responses are abolished by *α*7-nAChR specific antagonist, indicating that *α*7-nAChR is the primary mediator of nicotine and NNK signaling [58–60]. Similarly, serotonin and bombesin, the two autocrine growth factors, can activate the same signaling cascade* in vitro* [58, 59], whereas the effects of nicotine or NNK were abolished by a serotonin uptake inhibitor [59]. Nicotine or NNK induced DNA synthesis is effectively blocked by Ca2+ channel blockers [61]. In addition, NNK can cause ERK1-ERK2 dependent phosphorylation of m-calpains and *μ*-calpains and further promote the migration of small cell lung cancer (SCLC) cells [62]. The response can be blocked by ERK1-ERK2 specific inhibitors or RNAi silencing of calpains [62]. Furthermore, NNK can activate BCL-2 to inhibit apoptosis of SCLC cells, whereas PKC inhibitor staurosporine, ERK1-ERK2 inhibitor PD98059, or knockdown of* MYC* can block the effect [63].

The release of autocrine growth factors such as serotonin and mammalian bombesin is an important downstream response of *α*7-nAChR to stimulate the growth of cancer cells. In addition, several other autocrine growth factors of SCLC cells also activate the RAF1-ERK signaling pathway to cooperate with the *α*7-nAChR signaling cascade to stimulate the proliferation of cancer cells [64]. Consistently, inhibition of PKC or ERK1-ERK2 or upregulation of intracellular cyclic adenosine monophosphate (cAMP) can strongly suppress the nAChR-stimulated responses of SCLC* in vitro* [65, 66]. The suppression is probably mediated by inhibition of RAF1 by cAMP-dependent protein kinase A [67].

Heteromeric nAChRs are also expressed in non-small-cell lung cancers (NSCLCs); however, in smokers the nicotine or NNK responses are generally mediated by *α*7-nAChR as a result of desensitization of heteromeric receptors. Nicotine or NNK treatment of NSCLCs stimulates the proliferation and inhibits chemotherapy-induced apoptosis through activation of PI3K-AKT pathway and nuclear factor-*κ*B (NF-*κ*B) [44, 68]. Consistently, constitutive activation of AKT is observed in NSCLCs to promote resistance of apoptosis in chemotherapy [69]. And nicotine induced AKT-dependent upregulation of survivin and E3 ubiquitin-protein ligase (XIAP) to mediate the antiapoptotic response of NSCLCs [70]. In addition, *α*7-nAChR also mediates the activation of *β*-arrestin and protooncogene tyrosine-protein kinase Src (SRC) to promote the proliferation of NSCLC cells [71].

In immortalized human bronchial epithelial cells the downstream signal pathways activated by nAChRs include ERK1-ERK2 activated transcription factors, signal transducer and activator of transcription 1 (STAT1), NF-*κ*B, and GATA-binding factor 3 (GATA3). Interestingly, antagonist of *α*7-nAChR specifically blocked the stimulating effects of NNK, whereas antagonist of the heteromeric nAChRs specifically blocked the NNN responses [72]. nAChRs also control the release of growth factors such as proepidermal growth factor (EGF) in large airway epithelial cells; the effects are blocked by the selective antagonists of *α*7-nAChR through intervening with the Ras-Raf-ERK signaling cascade [73]. Thus the EGFR signaling pathway is incorporated into the nAChRs growth stimulatory effects in large airway epithelial cells.

The deregulation of nAChR subunits in primary lung cancer tissues is also evidenced by the epigenetic alterations of the nAChR genes [74–76]. Paliwal et al. reported that* cholinergic receptor*,* nicotinic*,* alpha 3* (*CHRNA3*) gene encoding the *α*3-nAChR subunit is frequently hypermethylated and silenced in lung cancer, and DNA methylation inhibitors can cause demethylation of* CHRNA3* promoter and reactivation of the gene [75]. Ectopic expression of *α*3-nAChR restored the protein level of the *α*3 receptor in H1975 lung cancer cell line and induced apoptosis [73]. They also observed a dramatic increase of Ca2+ influx response in the presence of nicotine elicited by knockdown of* CHRNA3* in *α*3-nAChR positive lung cancer cells, followed by activation of the AKT prosurvival pathway. Moreover, *α*3-nAChR depleted cells were resistant to apoptosis-inducing agents, underscoring the importance of epigenetic silencing of the* CHRNA3* gene in human cancer. Interestingly, they found* CHRNA3*, but not* CHRNA5*, is often hypermethylated and downregulated in cancer tissues, whereas a 30-fold upregulation of* CHRNA5* expression is observed in lung cancers compared with the normal lung [75]. Consistently, in a separate study *α*5-nAChR and *α*3-nAChR are identified as negative regulator of *α*7-nAChR mediated nicotine responses in human normal and bronchial cancer [21]. Knockdown of* CHRNA3* and* CHRNA5* in bronchial cancer cells and esophageal cancer cells leads to increased calcium influx induced by nicotine, which could be explained by the compensatory increase of the assembly of functional *α*7-nAChR on the cell membrane. Importantly, they have also identified downregulation of p63 after knockdown of* CHRNA5* or* CHRNA3*, which offered an explanation for the resistance to apoptosis in* CHRNA3* downregulated lung cancers. Moreover, knockdown of* CHRNA3* in A549 cells downregulates the cell-cell adhesion molecules and reduces the components of tight junctions (ZO-1) and adherens junctions (P120), analogous to epithelial cells undergoing epithelial-mesenchyme transition [77]. Together these findings suggest that *α*5-nAChR and *α*3-nAChR mediate the apoptotic responses and suppress the adhesion and migration of primary lung cancer cells and normal bronchial cells. In addition, the regulatory functions are mediated by the heteromeric (*α*3*β*2)2*α*5-nAChR rather than the AChR5 subunit alone.

## 6. nAChRs Signaling in Pancreatic Cancer

Cigarette smoking is most frequent risk factor associated with pancreatic cancer [78–81]. NNK can induce pancreatic cancer through the genotoxic effect of DNA adducts causing* RAS* gene mutations [40] but also has a hyperproliferative effect on pancreatic duct epithelia through *β*-adrenergic transactivation of EGF receptors [82, 83]. Recently, Al-Wadei et al. reported that nicotine and NNK promote the synthesis and release of adrenaline and noradrenaline to promote the proliferation and migration of pancreatic cancer cells [84]. And RNA knockdown experiments indicate the effect is mediated by *α*3-, *α*5-, and *α*7-nAChRs. Similarly, the process is coupled with increased *β*-adrenergic cAMP-dependent signaling and release of arachidonic acid in pancreatic cancer cell lines [45, 82, 85]. And the activation of CREB, ERK, SRC, and AKT pathways has been identified to mediate the oncogenic responses of nAChRs. Together these findings suggest nAChR mediated catecholamine synthesis, release, and transactivation of the EGFR signaling pathway promote the progression of pancreatic cancers.

Besides, nicotine/cigarette smoke promotes metastasis of pancreatic cancer through *α*7-nAChR mediated Mucin-4 (MUC4) upregulation. Chronic exposure to nicotine or cigarette smoke leads to increased expression of MUC4 in pancreatic cancer through activation of the *α*7-nAChR/JAK2/STAT3 and the MEK/ERK1/ERK2 signaling cascade [86]. And tobacco smoking induces chronic inflammation to trigger the development of pancreatic cancer [87]. The oncogenic effects of nAChR signaling in pancreatic cancer are also supported by the animal experiments, and N-nitroso compounds, formed from nicotine by nitrosation during the processing of tobacco plants, can cause pancreatic cancer in Syrian golden hamsters [88].

## 7. nAChRs Signaling in Oral and Esophageal Cancers

In oral and esophageal cancer, besides *α*7-nAChR, heteromeric nAChR composed of *α*3 and *α*5 subunits also regulates the responses of nicotine and NNK [15, 89, 90]. And chronic exposure to nicotine or tobacco smoke selectively upregulates *α*5-nAChR and *α*7-nAChR subunits in oral keratinocytes [55]. Similar to lung cancer cells [14], NNK preferentially binds to *α*7-nAChR with higher affinity, whereas NNN binds to heterometric nAChRs with higher affinity in oral and esophageal cancer cells. In esophageal carcinoma nAChRs mediated nitrosamine responses by activating signaling pathways such as Ras-Raf-ERK1-ERK2 and the JAK2-STAT3 pathway and NF-*κ*B and in GATA3 and STAT1 to promote the growth and inhibit apoptosis of the cancer cells [7].

Consistent with the neurotransmitters' effects on cancer, nAChRs mediated synthesis and release of adrenaline and noradrenaline are important downstream responses of nicotine stimulated growth of esophageal cancers. Consistently, increased proliferation of esophageal cancer cells is observed by adrenaline treatment, which is mediated by activation of Ras-MARK pathway and transactivation of EGFR [91, 92]. The mechanism is similar to the signal transduction mediated by nAChRs in colon cancer and pancreatic cancer [38, 93].

**Figure 2:**
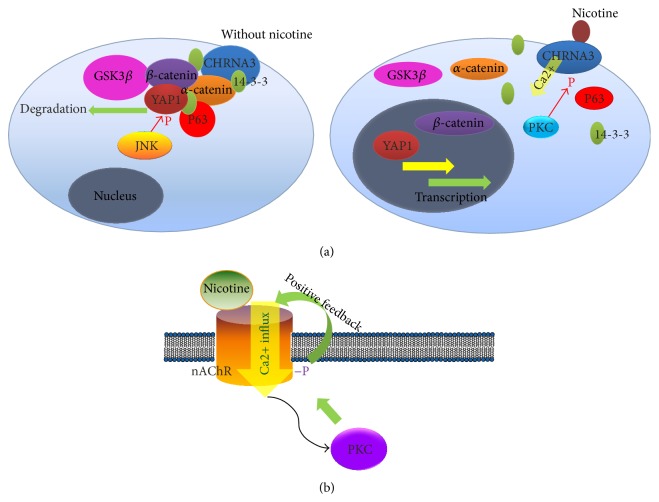
(a) Schematic model of nicotine action of YAP1 [94]. (b) Nicotine activates the positive feedback loop of PKC mediated phosphorylation of nAChR [97].

Other nAChRs mediated oncogenic signaling pathways are also implicated in esophageal cancer. Arredondo et al. reported that secreted mammalian SLURP1/SLURP2 are cell endogenous allosteric modulators of nAChRs signaling that enhance the responses of acetylcholine and trigger proapoptotic activity in human keratinocytes [89]. The expression of SLURP1 and SLURP2 is reduced in esophageal cancers, and exogenous expression of SLURP1 and SLURP2 in esophageal cancer cells reduced the colony forming ability of the cells in the presence of nitrosamine, also inhibiting the growth of NNK transformed keratinocytes in mouse xenograft. Recent work done by our group indicated nAChRs also mediated the nicotine activation of the oncogenic YAP1 of the Hippo signaling pathway in esophageal cancer, we also found upregulation of YAP1 in esophageal cancer samples is significantly associated with the smoking history of the patients, and the effects are regulated by PKC signaling, as PKC specific inhibitor can abolish the activation of YAP1 by nicotine treatment [94] (Figures 2(a) and 2(b)). Besides, nicotine promotes head and neck cancer through activation of endogenous FOXM1 activity by loss of heterozygosity involving the whole of chromosome 13 and copy number abnormality (CNA) in oral keratinocytes (KC) [53].

## 8. Conclusion Remarks

An increasing body of evidence suggests that nAChRs stay at the center of regulatory pathways of cholinergic and nicotinic signaling to regulate the growth and migration of the cells, also regulating angiogenesis of the endothelial cells during physiological and pathological conditions. In accord with the findings of multiple GWAS which indicate that SNPs of the gene cluster* 15q25*, which contains* CHRNA3*,* CHRNA5*,* CHRNB4*, are associated with increased risks of lung cancer and COPD as well as nicotine-dependence, recent cellular and molecular studies on nAChRs indicate that chronic exposure to nicotine or nicotine-derived carcinogenic nitrosamines upregulates the *α*7-nAChR and *α*9-nAChR and desensitizes the heteromeric *α*4*β*2-nAChR to activate the oncogenic pathways, promotes tumor angiogenesis, and inhibits drug induced apoptosis in multiple types of cancers. Although *α*7-nAChR is the oncogenic receptor responsible for most of the oncogenic responses in cancer, *α*9-nAChR has been shown to be upregulated in estrogen receptor positive breast cancer cells, and *α*9-nAChR stimulates the initiation and progression of breast cancer in coalition with estrogen receptor [95]. Collectively, these recent findings suggest that nAChR mediated oncogenic signaling plays an important role in the initiation and progression of cancer, which functions in parallel with the mutagenic and cytotoxic effects of tobacco smoke to promote the growth and angiogenesis of the tobacco related cancers. Thus nAChRs yield as promising new targets for the prevention, diagnosis, and treatment of tobacco related cancers.
